# Komatiites as Complex Adsorption Surfaces for Amino Acids in Prebiotic Environments, a Prebiotic Chemistry Essay

**DOI:** 10.3390/life12111788

**Published:** 2022-11-04

**Authors:** Abigail E. Cruz-Hernández, María Colín-García, Fernando Ortega-Gutiérrez, Eva Mateo-Martí

**Affiliations:** 1 Posgrado en Ciencias de la Tierra, Universidad Nacional Autónoma de México, Ciudad de México 04510, Mexico; 2 Instituto de Geología, Universidad Nacional Autónoma de México, Ciudad de México 04510, Mexico; 3 Centro de Astrobiología (CAB) CSIC-INTA, Carretera de Ajalvir km 4, 28850 Torrejón de Ardoz, Spain

**Keywords:** komatiite, amino acids, adsorption, concentration mechanism, prebiotic chemistry

## Abstract

Komatiites represent the oldest known terrestrial rocks, and their composition has been cataloged as the closest to that of the first terrestrial crust after the cooling of the magma ocean. These rocks could have been present in multiple environments on the early Earth and served as concentrators of organic molecules. In this study, the adsorption of five amino acids (glycine, lysine, histidine, arginine, and aspartic acid) on a natural komatiite, a simulated komatiite, and the minerals olivine, pyroxene, and plagioclase were analyzed under three different pH values: acid pH (5.5), natural pH of the aqueous solution of each amino acid and alkaline pH (11). Adsorption experiments were performed in solid–liquid suspensions and organic molecules were analyzed by spectrophotometry. The main objective of this essay was to determine if the complex surfaces could have participated as concentrators of amino acids in scenarios of the primitive Earth and if the adsorption responds to the change of charge of the molecules. The results showed that komatiite is capable of adsorbing amino acids in different amounts depending on the experimental conditions. In total, 75 systems were analyzed that show different adsorptions, which implies that different interactions are involved, particularly in relation to the type of amino acid, the type of solid material and the conditions of the medium.

## 1. Introduction

Earth has followed a very complex and fascinating history since its formation. Very soon after accretion and differentiation, igneous activity dominated the geological processes of the early planet [1]. Regarding the primeval crust formed by crystallization of the surface magma ocean in the Hadean [2], this phase in planet evolution provided conditions for both mantle differentiation and volatiles distribution [3]. Further evolution depended on processes such as volcanism, degassing, fractional crystallization, and fluid-rock interactions, among others [4]. The study of the formation and composition of this crust is relevant not only for understanding the early ages of our planet, but also for elucidating the origin of life itself [5].

There is no record of the Hadean rocks, which shaped the primordial crust [2]. Although there is a debate about the composition of primitive crust, it has been proposed that the primordial oceanic crust had, most likely, an extremely similar composition to rocks called komatiites, named after the Komati river in South Africa [6,7]. The term komatiite refers to an ultrabasic volcanic rock, whose lava probably erupted at temperatures above 1600 °C. Due to secular variations in major, minor and trace elements, along with stratigraphic compositional and textural differences, there is no accurate definition for these igneous rocks [8]. However, these rocks are “primitive” lavas and likely play a key role in the understanding of early Earth evolution. Komatiites could represent a primitive environment where chemical evolution reactions may have occurred [6].

Chemical evolution may have occurred in different environments on our young planet [9], including in oceans, lakes, lagoons, tidal pools, submarine hydrothermal systems [10], and microenvironments [11]. In primitive water bodies, chemical compounds, necessary for the creation of the first living beings, could have been dissolved and accumulated [12]. Nonetheless, the estimated concentration of organics in primitive oceans would be very low; according to some authors, a 10^−4^ mol·L^−1^ solution would result from the entire dissolution of all the available organic compounds in oceans [12]. Thus, free waters represent an exceedingly harsh environment in which to accomplish prebiotic reactions. However, at the interface of oceanic waters and minerals, from oceanic crust, the presence of these solids may have enhanced the concentration processes. In smaller water bodies, the concentration was likely higher, and reactions could have occurred in shallow microenvironments in which there was a variety of rocks and minerals. Hence, surfaces could have increased the concentration of organics in all aqueous environments.

Minerals have been proposed as concentration sites for organic molecules in primitive environments [13,14,15,16]. Indeed, many minerals were present on early Earth when prebiotic chemistry was accomplished; more than 500 minerals already existed on Earth at 4 Ga [17]. However, laboratory models of primitive environments have not yet included complex surfaces, such as those present in primitive crusts, either oceanic or continental.

On the other hand, amino acids have been used commonly in adsorption experiments related to prebiotic chemistry (for a review, refer to) [17,18]. Amino acids are essential organic molecules for living beings, being the building blocks of proteins, and they were very likely available in primitive environments either by its synthesis under the conditions of the primitive Earth or by its delivery in the primitive oceans via external sources [19]. For example, amino acids could have originated from Strecker synthesis from precursors, compounds available in the primitive ocean and in the atmosphere, such as HCN, NH_3_, aldehydes or ketones [20]. Also, it has been suggested that hydrothermal systems, with their temperatures and pH gradients, could have favored the synthesis of proteinogenic amino acids from hydrothermal fluids that contain H_2_, NH_3_ and H_2_S; the latter has been confirmed experimentally under the simulation of an alkaline hydrothermal environment [21]. Likewise, another suggestion is that organic molecules in primitive environments could have entered through exogeneous sources, such as meteorites, comets or interplanetary dust particles (IDPs) [10,22,23,24]. 

The concentration of amino acids that was maintained in the primitive oceans must have been dependent on the balance between their input into the environment, the internal synthesis and the degradation of amino acids due to physical factors in primitive ocean [20]. Also, due to the lack of geological evidence on the composition of the early ocean and atmosphere, estimates of the possible concentration of amino acids have been modified over time. For example, Dose [25] estimated that amino acids, synthetized from several energy sources, were in an approximate concentration of 10^−7^ mol·L^−1^ in the primordial ocean, synthetized from several energy sources. Stribling and Miller [26] calculated a concentration of amino acids of 3 × 10^−4^ mol·L^−1^, produced from a mixture of precursors in a simulated atmosphere and promoted mainly by sparks. Cleaves et al. [20] more recently consider that the concentration of these molecules should have been between 0.2 mol·L^−1^ (as the upper limit) and 3 × 10^−4^–3 × 10^−19^ mol·L^−1^ (as the lower limit); their estimates depend on the amount of N in the Earth’s crust, the oceanic reservoir, and on the production of amino acids in hydrothermal systems. Similarly, calculations have been made to estimate the contribution of amino acids from exogeneous sources. Sugahara and Mimura [23], based on comet impacts simulations, estimated the delivery of glycine to the early Earth to be 4 × 10^−10^ mol·L^−1^ and 1 × 10^−8^ mol·L^−1^ using the Lunar Cratering Model and the Nice Model, respectively. The general agreement among the scientific community has been that the concentration of amino acids in the early ocean must have been low. Thus, concentration mechanisms in primitive scenarios were extremely necessary. In these environments, other concentration mechanisms could have existed; however, adsorption by mineral surfaces is suggested to have been the most widespread mechanism on prebiotic Earth [19].

In this study, a first attempt to determine the ability of complex surfaces to adsorb amino acids was essayed. The solids employed as sorbents were komatiite, plagioclase, pyroxene, olivine, and a mixture including the most abundant minerals in those rocks (a compositional model for primitive oceanic crust). 

As organics, proteinogenic amino acids (glycine, lysine, histidine, arginine and aspartic acid) were selected, whose abiotic synthesis (under simulated conditions of the primitive Earth, or the interstellar medium) or whose detection (in meteorites or interstellar medium) has been reported in the literature [19,27,28,29,30]. Histidine and arginine have been synthesized abiotically under different experimental conditions as mentioned above, but they have not been found on carbonaceous chondrites [31]. However, it has been observed that lysine, arginine, and histidine are important binding domains and contribute to the stabilization of catalytic conformations of ribozymes, which makes them important molecules in the origin of life [31]. In addition, it has been observed that they (lysine, histidine, and arginine) condense more extensively than non-proteinogenic amino acids (ornithine; 2,4-diaminobutyric acid; and 2,3-diaminopropionic acid); they are more selective in oligomerization because of the nature of their side chains, which represents an advantage for the formation of more complex molecules [32].

Due to their chemical structure, amino acids hold both a basic (-NH_2_) and an acidic group (-CO_2_H) in their structure. In aqueous solution, these groups ionize and, in general, the basic group has a p*K*a between 9 and 10, though acid p*K*a is close to 2; this implies that, at almost any pH value, amino acids exist as ions: cation (NH_3_^+^), anion (COO^−^) or zwitterion form (NH_3_^+^, COO^−^). This behavior is fundamental in the study of amino acid/mineral interactions. For this reason, a series of experiments was designed, including pH changes of the solution, as a determinant variable, for the sorption of amino acids. The main goal of this study was to determine whether complex surfaces could have actively participated in the adsorption of amino acids in different scenarios of primitive Earth. We focus on determining the best scenarios for amino acid adsorption onto those surfaces as a function of molecules charge change.

## 2. Materials and Methods

### 2.1. Materials 

All experiments and procedures were realized with ultrapure water (Milli-Q, Millipore, Burlington, MA, USA). The olivine sample used in this work was the only one that was obtained commercially as peridote. The other minerals and the rock used were natural samples collected in the field and obtained through the donations of Professors Luca Ferrari (CGEO-UNAM), Fernando Ortega and Antoni Camprubí (IGl-UNAM). The chemical reagents used were purchased from Sigma-Aldrich Inc^®^., St. Louis, MO, USA.

### 2.2. Methods

#### 2.2.1. Cleaning of Geological Material 

Geological materials were cleaned individually. Samples were fragmented into small pieces no larger than one centimeter. All the fragments were weighed and deposited into a beaker. 10 mL of all the solutions for each gram of solid was used. A 3% KNO_3_ solution was prepared and added to the geological material, and then the mixture was put into an ultrasonic bath for 30 min. After this process, the sample was decanted, and the material was rinsed with pure water. To eliminate all the rest of the solution, the material was stirred with enough pure water for 30 min, and then the water was poured out. A 3% HNO_3_ solution was prepared and added to the material. An ultrasonic bath was conducted for 30 min. Subsequently, the solution was decanted, and the fragments were rinsed with pure water. Finally, enough water was added and stirred for 30 min. Once this step was concluded, the water was drained, and the geological material was maintained in the same beaker for drying at room temperature.

After the cleansing of geological materials, they were milled in an agate mortar and sifted. Particles <125 µm were used for all experiments.

#### 2.2.2. Characterization of Komatiite Sample by X-ray Diffraction Analysis 

Minerals phases in komatiite were determined by X-Ray Diffraction (XRD). Milled samples of rocks (particles < 75 µm) were placed in an aluminum sample holder. Measurement was made in an angular range 2θ (2 Theta) from 5° to 80° with a step of 0.003° and an integration time of 40 s per step. An Empyrean^®^ Diffractometer with a nickel filter, fine focus copper tube, monochromator, and PIXcel3D detector was employed. This analysis was carried out at Laboratorio de Difracción de Rayos X, of the Laboratorio Nacional de Geoquímica y Mineralogía (LANGEM-IGl-UNAM). 

#### 2.2.3. Estimation of the Point of Zero Charge of Geological Materials 

The point of zero charge (*pzc*) of each geological material was determined according to de Souza et al. [33] 100 mg of mineral or rock and 250 µL of either a KCl (1 mol·L^−1^) solution or pure water were mixed. After 24 h, pH values were measured with a potentiometer Thermo Scientific Orion Versa Star Pro^®^. The process was conducted in triplicate for each geological material. The *pzc* values were calculated with the following equation:

pH*_pzc_* = 2(pH_KCl_) − pH_H2O_ (1)

#### 2.2.4. Quantification of Amino Acids

Glycine (Gly) (hydrophobic aliphatic), and the L-amino acids lysine (Lys), histidine (His), arginine (Arg) (the three hydrophilic basic), and aspartic acid (Asp) (hydrophilic acid) were selected; their principal chemical properties can be consulted in Table 1. These amino acids were quantified by UV-vis spectrophotometry through a colorimetric derivative, by the ninhydrin method [34]. According to this method, ninhydrin reacts with the amino group and gives a colored derived compound. The derivative is then measured by visible spectrophotometry at 570 nm. A Varian^®^ Cary 100 Scan spectrophotometer was used to measure the absorbance. A calibration curve for each amino acid was made for quantification.

#### 2.2.5. Effect of pH Variation 

Three pH values were used in the experiments: (i) the natural pH value of each amino acid in an aqueous solution (which value is reported in Table 2); (ii) acidic pH (value 5.5) adjusted by the addition of an acetate buffer; and (iii) alkaline pH (value 11) modified by the addition of an NaOH solution (0.1 mol·L^−1^).

#### 2.2.6. Experimental Systems

A komatiite sample and different samples of the main forming minerals of this rock (i.e., olivine, pyroxene, and plagioclase) were used. The purpose was to determine if any of these mineral phases had more of an effect on the adsorption processes. Since our komatiite sample presents alterations, we decided to use a mixture of the major minerals present in komatiites in approximately the same proportion that they would have occurred in an unaltered rock (olivine 49.29%, plagioclase 29.57%, and pyroxene 21.12%), as a compositional simulated komatiite, and hence a simulation of a primordial oceanic crust. Therefore, 75 different systems were studied, considering pH changes in a solution. All of them were treated with the same experimental adsorption procedure.

The experiments were carried out with the amino acids individually, and no mixtures of these were made. Under ideal conditions, the adsorption of amino acids is dependent on the value of the isoelectric point, when adsorbed by electrostatic interactions on a surface; thus, amino acids are expected to be selectively adsorbed. However, it is likely that when mixtures of amino acids are used, they could interact with each other, which scatters this ideality; therefore, the adsorption of certain amino acids in a mixture would depend on the presence of other amino acids, which modifies the behavior of adsorption [18]. Additionally, in mixtures, polymerization between them is also feasible, since intermolecular chemical forces drive the dynamics of these organic compounds towards self-recognition and assembly [35]. To avoid interactions between amino acids and to maintain the prebiotic context, a low concentration of amino acids was used. Also, when water is present, it has been observed that there is competition between the formation of H bonds that link amino acids (amino acid_1_-amino acid_2_) or amino acid and water molecules around it [35].

#### 2.2.7. Adsorption Experiments 

Solutions of each amino acid (1 × 10^−4^ mol·L^−1^) were prepared in ultrapure water (Milli-Q, Millipore). 5 mL of each amino acid solution were used, mixed with 100 mg of each geological material [20:1 solid-solution ratio (mg/mL)]. These suspensions were content in polyallomer centrifuge tubes and shaken for 24 h onto a multi-tube platform Thermo ^®^. This duration was selected because in prebiotic chemistry, many experiments are performed considering 24 h as a suitable time to study sorption [36]. The suspensions were centrifuged using a Beckman Coulter ^®^ Allegra 64R Centrifuge for 35 min at 36,000 rpm. The supernatant was recovered and analyzed by the ninhydrin method. With the calibration curves, the remaining concentration in the supernatant of each sample was determined. All experiments were conducted in triplicate, at room temperature (22 °C). 

## 3. Results

### 3.1. Characterization of Geological Materials 

#### 3.1.1. Mineral Phases in Komatiite Sample 

The results of XRD analysis can be observed in Table 3, and through this information, we determined that in the komatiite sample, just 10% of the total is fresh olivine, and a larger quantity is lizardite, a type of serpentine, which is evidence of an altered komatiite. In this rock, olivine is altered into hydrated minerals such as chlorite or serpentine, and those alterations are characteristics of the environment in which komatiites are generated [37]. Other mineral phases found in our komatiite sample were pyroxene, plagioclase, and other phyllosilicates.

#### 3.1.2. Point of Zero Charge

The results of estimation of *pzc* of all geological materials, including the mix used as a simulation of an unaltered komatiite (komatiite-simulated), are shown in Table 4. The value of point of zero charge of olivine was found at 9.9, for pyroxene at 7.5, and in the case of plagioclase the value obtained was 8.9. The *pzc* value for the komatiite was 8.2, and it was observed that the simulated komatiite showed a higher value (10.01) compared to the natural rock.

### 3.2. Adsorption Experiments 

**Figure 1:**
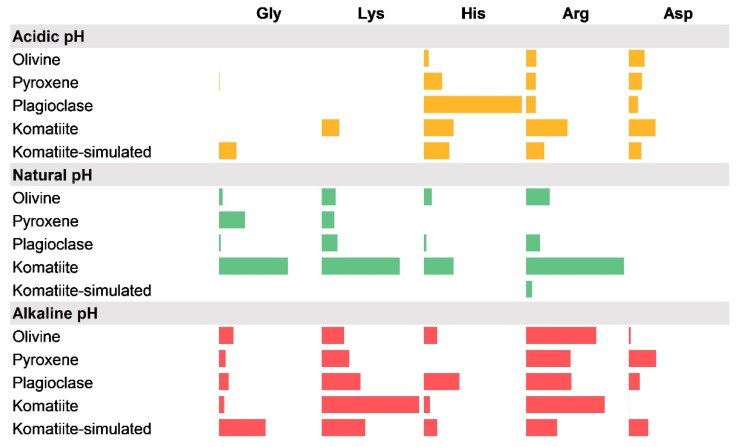
A graphic representation of the amount of amino acids adsorbed on different surfaces. The bar is proportional to the amount adsorbed. Three pH values are shown: acidic pH (5.5) in yellow; natural pH of the solution (see Table 2) in green, and alkaline pH (11) in red. The results are shown in mmol of amino acid adsorbed per mg of solid (see Table 5, Table 6 and Table 7).

Results are shown as each system; each one is the combination of one mineral or geological material, and one amino acid at a specific pH value; these results can be observed in Figure 1. Adsorbed concentrations are presented in mmol of the adsorbed amino acid per milligram of solid used. 

#### 3.2.1. Adsorption Experiments at Natural pH Values 

Natural pH refers to the pH obtained by dissolution of the amino acids in water, without further modifications. At this condition (natural pH of the amino acids) in 15 of the 25 systems, there was the adsorption of the organics onto minerals, the results of which are shown in Table 5. In the case of olivine, four of the amino acids were adsorbed; the amino acid with the highest adsorption in olivine was arginine with 2.11 × 10^−4^ mmol/mg, while the rest of amino acids present lower adsorption: lysine (1.27 × 10^−4^ mmol/mg), histidine (7.17 × 10^−5^ mmol/mg), and glycine (3.18 × 10^−5^ mmol/mg). For pyroxene, only two amino acids were adsorbed onto this mineral, glycine with 2.31 × 10^−4^ mmol/mg and lysine with 1.12 × 10^−4^ mmol/mg. In the case of plagioclase, lysine was the most adsorbed amino acid with 1.41 × 10^−4^ mmol/mg, and then arginine with 1.22 × 10^−4^; the other two amino acids adsorbed onto plagioclase were histidine (2.15 × 10^−5^ mmol/mg) and glycine (1.55 × 10^−5^ mmol/mg), but to a lesser extent. The adsorption of lysine and arginine onto komatiite was observed, 6.99 × 10^−4^ mmol/mg and 8.76 × 10^−4^ mmol/mg, respectively; in addition, glycine (6.14 × 10^−4^ mmol/mg) and histidine (2.64 × 10^−4^ mmol/mg) were also adsorbed. In the case of the mixture of minerals, as a simulated-komatiite, only arginine was adsorbed (5.14 × 10^−5^ mmol/mg). Acid aspartic was not adsorbed in any of the systems at a natural pH of the solutions.

#### 3.2.2. Adsorption Experiments at Acidic pH 

This section may be divided by subheadings, which should provide a concise description of the experimental results, their interpretation, and the experimental conclusions that can be drawn. Table 6 shows all the quantities adsorbed in the experimental systems. 

A vastly different behavior was witnessed in the sorption experiments at an acidic pH value, in comparison with the results mentioned above. Onto olivine, only three amino acids were adsorbed: aspartic acid (1.52 × 10^−4^ mmol/mg), arginine (9.55 × 10^−5^ mmol/mg), and histidine (4.52 × 10^−5^ mmol/mg), while in the pyroxene sample the amino acid with the highest adsorption value was histidine (1.75 × 10^−4^ mmol/mg). Other amino acids adsorbed were aspartic acid (1.27 × 10^−4^ mmol/mg), arginine (8.93 × 10^−5^ mmol/mg) and glycine (4.74 × 10^−6^ mmol/mg). In the case of plagioclase, the same three amino acids as for olivine were adsorbed (aspartic acid, arginine, and histidine); histidine was the one with the highest value of 9.40 × 10^−4^ mmol/mg. Finally, aspartic acid and arginine showed very similar adsorption amounts, 9 × 10^−5^ mmol/mg and 8.93 × 10^−5^ mmol/mg, respectively, on plagioclase.

In the case of adsorption onto komatiite, four of the amino acids were adsorbed: histidine (2.85 × 10^−4^ mmol/mg), aspartic acid (2.57 × 10^−4^ mmol/mg), and lysine (1.69 × 10^−4^ mmol/mg), while arginine was the molecule with the highest adsorption (3.96 × 10^−4^ mmol/mg). For the simulated-komatiite systems, four amino acids were also adsorbed: histidine (2.44 × 10^−4^ mmol/mg), arginine (1.72 × 10^−4^ mmol/mg), aspartic acid (1.21 × 10^−4^ mmol/mg), and glycine (1.65 × 10^−4^ mmol/mg), that, on this surface, was adsorbed at an acidic pH. In comparison, from the experiments at a natural pH, aspartic acid was adsorbed in all geological materials.

#### 3.2.3. Adsorption Experiments at Alkaline pH

The results of the experiments at alkaline pH can be observed in Figure 1 and Table 7. There was a response for 23 of the 25 systems studied, in contrast with both pH values presented above; in fact, the alkaline pH was the one in which adsorption response was obtained for most of the systems. All the amino acids were adsorbed on olivine; arginine was the molecule with the highest value at 3.4 × 10^−4^ mmol/mg; the remaining amino acids were adsorbed in lower amounts—lysine (1.08 × 10^−4^ mmol/mg), glycine (6.90 × 10^−5^ mmol/mg), histidine (6.52 × 10^−5^ mmol/mg), and aspartic acid (1.04 × 10^−5^ mmol/mg). For the systems where pyroxene was used, the highest adsorption was for arginine with 2.15 × 10^−4^ mmol/mg, while, successive to this, lysine and aspartic acid with 1.35 × 10^−4^ mmol/mg and 1.34 × 10^−4^ mmol/mg, respectively, were found, and, finally, glycine (3.18 × 10^−5^ mmol/mg); histidine was not adsorbed in the pyroxene sample. In the case of plagioclase, all amino acids were adsorbed, and arginine was the amino acid with the highest sorption, 2.19 × 10^−4^ mmol/mg; the values of sorption for the rest of the amino acids were: lysine (1.9 × 10^−4^ mmol/mg), histidine (1.72 × 10^−4^ mmol/mg), aspartic acid (5.29 × 10^−5^ mmol/mg), and glycine (4.68 × 10^−5^ mmol/mg). In the case of komatiite, the molecules with the highest adsorption were lysine (4.76 × 10^−4^ mmol/mg) and arginine (3.82 × 10^−4^ mmol/mg); histidine and glycine were also adsorbed, with 2.91 × 10^−5^ mmol/mg and 2.39 × 10^−4^ mmol/mg, respectively. Compared with the natural rock, the komatiite-simulated sample mostly adsorbed glycine (2.27 × 10^−4^ mmol/mg), followed by lysine with 2.12 × 10^−4^ mmol/mg; the lowest values of adsorption were for aspartic acid (9.57 × 10^−5^ mmol/mg) and histidine (6.45 × 10^−5^ mmol/mg), and finally arginine was adsorbed in 1.5 × 10^−4^ mmol/mg. 

#### 3.2.4. Adsorption Trends under Different pH Values

**Figure 2:**
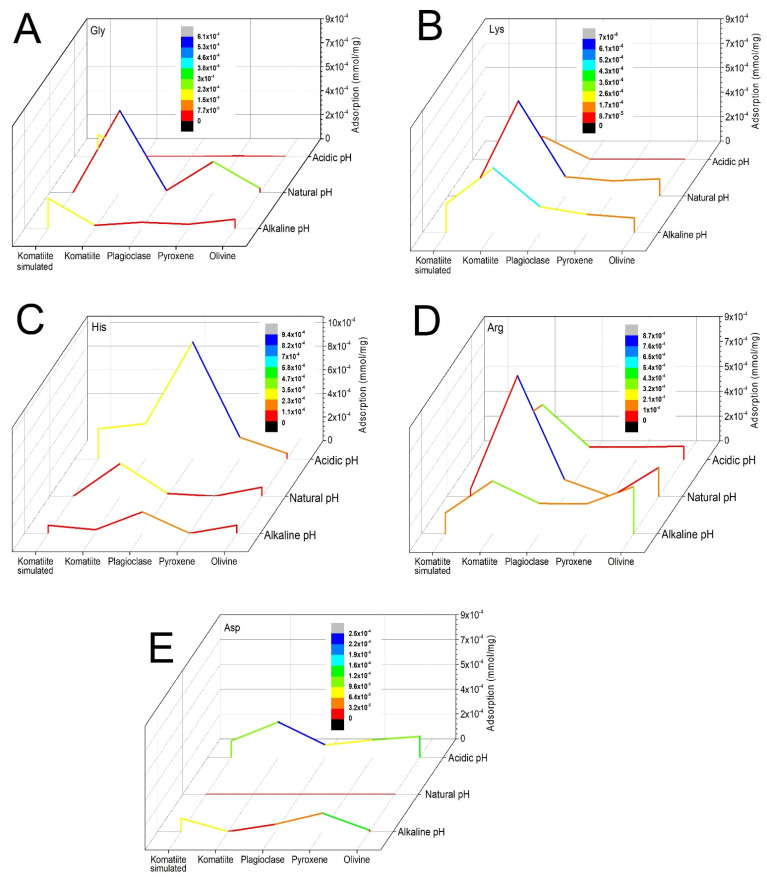
A comparison of the amounts adsorbed from each system at the three pH values used in the experiments: acid pH (5.5), natural pH of the solutions (see Table 2) and alkaline pH (11). (**A**) All glycine (Gly) systems; (**B**) all lysine (Lys) systems; (**C**) all histidine (His) systems; (**D**) all arginine (Arg) systems; (**E**) all aspartic acid (Asp) systems.

As can be seen in Figure 2, if the adsorbed quantities of each amino acid in each geological material are compared at the natural pH of the solution and with the changes of pH to acid and alkaline, the pH value does indeed influence the adsorption.

In general, the adsorption of glycine (Figure 2A) was higher using the natural pH of the solution. The acid pH did not favor the adsorption of this amino acid and when the alkaline pH was used, the highest amount adsorbed was in the system with the simulated komatiite. Lysine (Figure 2B) is mostly adsorbed at a natural and alkaline pH in the komatiite system. It was observed that the acidic pH considerably favored histidine (Figure 2C) adsorption in all materials except for olivine, which maintained low amounts of adsorption in all cases. The arginine (Figure 2D) adsorption was favored using alkaline pH for most of the cases; however, it was observed that the adsorption was higher in the komatiite system at both natural and alkaline pH. The response of aspartic acid (Figure 2E) was very clear when using the natural pH; no adsorption was observed, and it showed the tendency to be adsorbed in greater amounts under acidic conditions.

In addition, it can be observed that for glycine, lysine, arginine and aspartic acid, komatiite was the system that had the highest adsorption at different pH values. In the case of histidine, the highest adsorption was recorded in plagioclase at acidic pH.

## 4. Discussion

### 4.1. Mineral Phases in Komatiite Sample

The mineralogy of an unaltered komatiite is mainly composed of Mg-rich olivine crystals due to the magma from which these rocks are formed; those olivine crystals constitute the skeleton of the characteristic spinifex texture of these rocks; the matrix in which olivine crystals are embedded is commonly composed of minerals such as pyroxenes, chromite, and plagioclase [37,38]. The komatiites that belong to the Gorgona Islands complex have been described as young komatiites (98.7 ± 7.7 to 64.4 ± 5 Ma) [39] whose spinifex texture is composed of spicules made of olivine forsterite-type and pyroxene with high calcium content and low aluminum values. In addition, andesite-type plagioclase and chloritized and vitrified glass have been found in the mineral composition of these komatiites from the Gorgona complex [40]. These mineral phases mentioned above are consistent with the mineralogical composition found by X-ray diffraction analysis performed in this study for our komatiite sample. Also, it has been described that komatiites suffer alterations in their original mineral phases due to the environment where they are found. In this process, olivine undergoes alteration to hydrated minerals such as serpentine [37]. Specifically for the komatiites of the Gorgona complex, it was observed that the rock-forming minerals are usually transformed into minerals such as chrysotile, lizardite, magnetite or chlorite [41]. The mineral phases that we found in the komatiite sample are consistent with the alterations reported in the literature for Gorgona komatiites. The low percentage of fresh olivine cores (10%) that was found is indicative that the sample has a high level of alteration, mainly due to the serpentinization of the olivine crystals.

### 4.2. pzc Estimations

One of the most important parameters in studies using solid-aqueous interfaces is the *pzc*, the pH value at which the surface charge of the solid equals zero. This value is important because in these interfaces the surface charge can be modified if the conditions of the medium change—the surficial charge—is pH-dependent, due to the presence of chemical species and ions that can exist in a solution [42]. The *pzc* value is widely used for identifying interactions through electrochemical forces between the surface and medium which includes the molecule in a solution [43].

It has been observed that *pzc* values published in the literature, for the same materials usually present variations, could be the result of the different methods used to estimate the *pzc* [44]. Moreover, these variations are governed by the nature of the materials, whether they are natural or synthetic samples, since properties such as the crystalline structure and the state of hydration of the material determine the adsorption capacity of ions on the surface, and these properties are different between synthetic and natural materials [45]. Nevertheless, what contributes the most to the variation in the *pzc* values, and the most important factor to consider, is the purity of the materials studied [46]. Hence, the fact that in this study only natural samples of all minerals and rocks have been worked with is probably the reason why the values obtained and presented in Table 4 show differences compared to other estimations.

The *pzc* values of olivine obtained in this study (9.9) were very close to those reported in the literature. Luce and Parks (1973) [43] reported the *pzc* value of forsterite-type olivine from a natural sample without alterations in 8.9, and Pokrovsky and Schott (2000) [47] estimated the pH value of *pzc* of a natural sample of forsterite by titration in 10. However, the *pzc* obtained for pyroxene (7.5) was discordant from those reported for this mineral. Lasaga (1984) [48] mentioned the *pzc* value for pyroxenes in a range of 2.6–3.8; therefore, the result we obtained was found to be above the reported range. No references were found to compare the point of zero charge obtained for the komatiite and the mixture of minerals that constitutes the simulated komatiite, since it is not common to make these measurements in rocks.

### 4.3. Adsorption of Amino Acids in Geological Materials

It has been observed that the adsorption of a molecule onto a surface is highly dependent on three fundamental aspects: the characteristics of the organic molecule, the properties of the surface, and the medium [49]. Here, the results obtained from the adsorptions for the systems used, based on the first two aspects mentioned, will be discussed. The medium relevance will be discussed later, in the next section.

#### 4.3.1. Characteristics of the Amino Acids

In the first instance, the adsorption process can occur due to the differences in charges between the chemical functional groups of the organic molecules and the group that substrates expose on the surface. Theoretical studies of the adsorption of amino acids onto silicates can be found in the literature, and these describe an atomic level mechanism by which adsorption can be carried out. For example, Lomenech et al. [50] computationally analyzed the interaction of glycine on amorphous silica simulated by isolated silanol groups from the gas phase. Through the calculations carried out, they suggest that the amino acid preferentially interacts through the terminal -COOH group as an H acceptor, and with the silanol groups as an H donor, forming non-covalent interactions. In another approach, Escamilla-Roa and Moreno [51] studied the adsorption of glycine (in the gas phase) on forsterite via computational modeling and discovered different types of interactions: from H bonds to oxygen transfer between the amino acid and the top of the surface, depending on whether the glycine was in a neutral or zwitterionic form and whether the surface was dipolar or non-dipolar. These approximations are very useful in understanding the mechanisms of amino acid adsorption at the atomic level; nonetheless, it has been observed that the interactions between amino acids and mineral surfaces are different in the aqueous phase in respect to the gas phase [49]. At low organics concentrations, in an aqueous medium, the interactions through water molecules must be a relevant factor, since possible interactions between molecules of the same amino acid are avoided [52]. In an aqueous solution, amino acids change from a neutral to a zwitterionic form where they are solvated and present characteristics of divalent ions [53]. Water molecules in an aqueous medium are an important factor for the adsorption. When the solvation sphere is formed, zwitterionic amino acids are stabilized by water molecules, and this is a more stable structure (in the process of adsorption on a silicate surface) than one of the amino acids in neutral form. To continue with the stability of the solvation sphere of amino acids in the solid/liquid interface, the silanol groups on the top of the surface substitute one or more water molecules and establish physisorption-type interactions [54]. The silanol group (Si-O-H), present in all silicates, is commonly related to the interaction and retention of organic compounds, therefore acting as a surface adsorption site [55].

Due to the above information and to the fact that all the amino acids present a carboxylic acid (COOH) and an amino group (NH_2_), both available to interact with the solid phases, we would expect that all the systems at the natural pH of the solution would have similar adsorption values, if the adsorption mechanism only depended on the amino and carboxyl groups. The results show that the systems presented high variations and differential adsorptions between each of them, suggesting that the adsorption must be largely governed by the possible interactions of the side chain, specific to each amino acid. On the one hand, the water molecules solvate the carboxyl and amino groups of the amino acids; the H atoms are oriented toward the COO^−^ and the O atoms are oriented toward the NH^3+^ group. 

On the other hand, amino acids with long side chains including CH_2_ groups, such as arginine and lysine in this case, can generate hydrophobic interactions with other charged groups and with water molecules. It has been observed that these interactions tend to increase with growing chain length of the amino acid side chain, and the presence of a polar terminal functional group increases the number and intensity of possible interactions with other molecules, groups, or charged ions, and with water [56]. This type of interaction in amino acids with a longer side chain can be considered an explanation for the fact that lysine and arginine were adsorbed more than the other amino acids in the adsorption experiments at the natural pH of the solutions.

**Figure 3:**
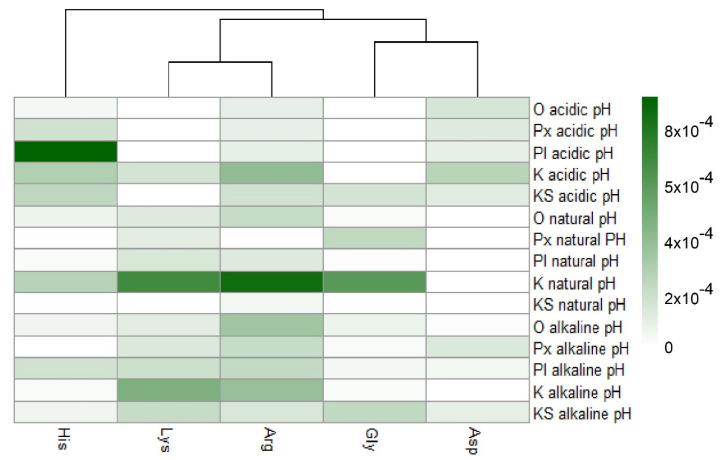
A heatmap showing the general ordering of the adsorptions presented by the different amino acids used. It is shown how aspartic acids and glycine are grouped by their similarity, and in another group arginine and lysine are clustered. In dark green, the highest values of adsorption are shown, and in light colors the lowest values are shown. Row names: O = olivine, Px = pyroxene, Pl = plagioclase, K = komatiite, and KS = komatiite-simulated.

As shown in Figure 3, histidine was the amino acid with the highest adsorption values. With this result, the unique structure of histidine can have a relevant weight. Histidine holds an imidazole group, as a side chain, that gives the amino acid (His) the ability to coordinate with metal cations and to be an acceptor or donor of H in H-bonds, even in different pH conditions [57]. Arginine and lysine were arranged into a subgroup due to their similar adsorption values; both are basic amino acids and have four carbons each in their side chains. In another subgroup, aspartic acid and glycine are found to be amino acids with similar adsorption values, and there the amino acids were adsorbed in smaller amounts. In general, a difference can be determined between amino acids with a 1:1 ratio of the carboxylic acid-amino group and the ones with different proportions. The presence of an extra amino group apparently increases adsorption, such as in the case of arginine and lysine. 

In addition, it is known that functional groups, located on the side chains of many amino acids that shape proteins, are capable of forming complexes with metal ions; these groups are binding sites for metals in proteins, playing an important role in the catalytic activity of enzymes. For example, the imidazole group of histidine and the β-carboxyl group of aspartic acid are side chains capable of binding to metal centers [58]. In nature and especially in the Earth’s crust, minerals containing metallic elements in their structure are very common. Silicates are the most abundant minerals in the crust; they are mainly composed of oxygen and silicon, but contain other elements (such as aluminum, iron, magnesium, sodium, potassium, and calcium) that are found in abundance and combined with silica to form 99% (wt) of the mass of the crust [59]. In the context of the early Earth, after magma cooling, the early Hadean crust would have been comprised of basalts and komatiites; these rocks are themselves comprised of olivine, pyroxene, magnetite, spinel, augite, and plagioclase minerals [60]. Therefore, the presence of metallic elements must have been very common on the surface of the Earth’s early crust, and this sort of interaction between the lateral chain of amino acids and metals was possible. Nonetheless, in the case of aspartic acid, the adsorption at the natural pH of the solutions was null for all the geological materials, so the interactions between the β-carboxyl group of aspartic acid and the metallic elements of the surfaces are not fulfilled in our experiments.

#### 4.3.2. Properties of the Geological Materials

The characteristics of the surface are another fundamental factor in sorption processes. In several works, the synthetic silica surface is widely used to simulate the surface of silicates. For example, Gao et al. [61] studied the adsorption of arginine, alanine, leucine, phenylalanine, and glutamic acid onto a mesoporous pure silica SBA-15. They observed that in this material the adsorption processes are governed by two types of interactions: (1) electrostatic forces between the ionic forms of amino acids in a solution and the positive (≡SiOH_2_^+^) and negative charges (≡SiO^−^) present on the surface; and (2) the formation of hydrogen bonds between amino acids and water molecules; the possible formation of hydrogen bonds between the amino acids (in ionic form) and the ≡SiOH groups are neglected. Undoubtedly, these works help us to understand the interactions between organic molecules and surfaces, but they present a homogeneity that natural geological materials do not show. The geological materials employed here were pulverized to increase the surface in which the amino acids can interact, but it was also sought to homogenize the natural samples, and they cannot be completely homogeneous, especially rock samples. Despite this, at the microscopic level, minerals have surface irregularities that generate variations in the adsorption of organics, such as amino acids. This effect was proposed by dos Santos et al. [52] after studying the adsorption of amino acids in 11 minerals of different nature, including forsterite-type olivine, two different pyroxene samples, and a basaltic lava. Corroboration of this proposal is the study of Galvez–Martinez et al. [62], who analyzed the adsorption of glycine on monocrystalline pyrite (100) by XPS and observed that the atomic ordering of the surface determines the form as the amino acid is adsorbed on the surface. 

**Figure 4:**
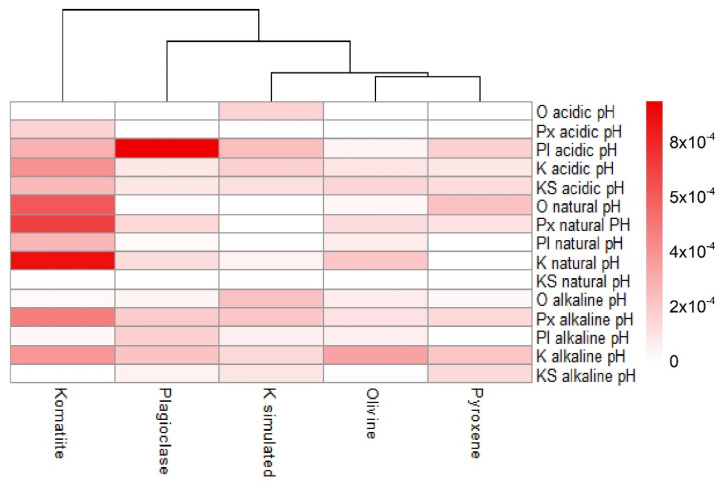
A heatmap showing the ordering of the surfaces by the amount of the adsorbed amino acids. In dark colors the systems that obtained the highest adsorption, in light colors those that presented the least adsorption. Komatiite-simulated is represented in the figure as K simulated.

As can be seen in Figure 4, the surfaces adsorb amino acids in different ways. In the heatmap, olivine and pyroxene are grouped in a subgroup, since they present similar adsorption values. The other geological materials are not grouped. The hierarchical order of adsorption was: komatiite, plagioclase, simulated komatiite, olivine, and pyroxene from highest to lowest adsorption. The high adsorption of certain amino acids on komatiite suggests that complex surfaces (including a matrix and various mineral phases embedded in it and organized in a particular way) may favor sorption. 

There is a high probability that other aspects, such as the superficial area of the materials and their chemical composition, influence the adsorption. However, these aspects were not studied in this work and their possible contributions go beyond what can be discussed in this manuscript; without a doubt, these aspects need to be considered for future work.

### 4.4. Adsorption of Amino Acids in Geological Materials under Different pH Values

In the context of prebiotic chemistry, the role of pH has been highly discussed in the literature. The exact composition and values of parameters such as ionic strength or composition of primitive ocean are uncertain. Therefore, several pH values of those oceans have been proposed. Proposals are based on different processes and conditions, either by the ions-interchange present [63], by the interactions between the rocks of the primitive crust and the water [60], the primitive oceans seawater composition [64] or by more local environments, such as conditions prevailing on hydrothermal systems [65], to mention a few. In this work, three different pH values were used that could be representative of various environments of the early Earth.

As mentioned before, another fundamental aspect in the adsorption process is the medium in which this phenomenon occurs. In the first instance, the simplest form of adsorption occurs due to differences in charges between the surface and the organic molecule. The charges generated on the surfaces of solids depend highly on the pH of the medium. In this essay, we study the pH change in the suspensions as a variable that may or may not favor the adsorption of glycine, lysine, histidine, arginine, and aspartic acid on the five geological materials tested. The surface is positively charged when the pH value is below the *pzc* value and above this point, the surface is negatively charged [61]. Correspondingly, the chemical form of amino acids changes with the pH value due to their ionizable groups. When the pH value of the medium is equal to the isoelectric point, the molecule is electrically neutral [66]. Therefore, at that last pH value, interactions through electrostatic forces are not expected. 

**Figure 5:**
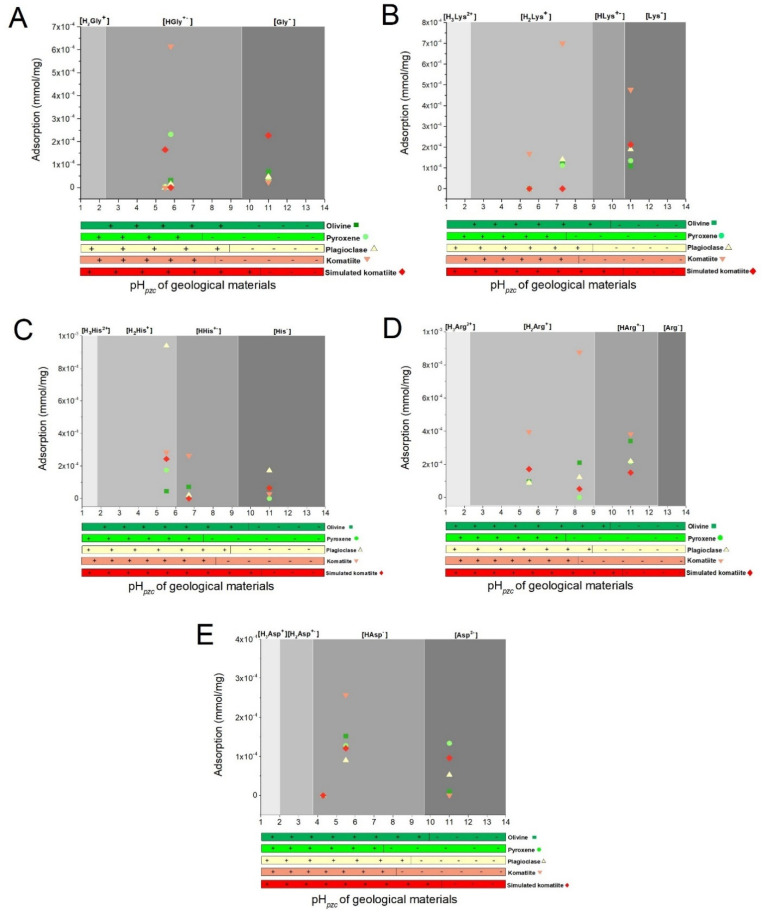
Amounts of each amino acid adsorbed in the geological materials at the three different pH values used. In addition, the distribution diagram of species of each amino acid can be observed and in the lower part the values obtained from the point of zero charge (*pzc*) of the geological materials. This figure shows the charge of the amino acid and the surface charge of the solids to observe if the adsorption is mainly directed by electrostatic interactions. (**A**) glycine; (**B**) lysine; (**C**) histidine; (**D**) arginine and (**E**) aspartic acid.

Gao and collaborators [61] reported the increase in the adsorption of arginine on pure silica as pH increases, for values above 2.5—results that agree with those obtained in this work. The increased adsorption of arginine at more alkaline pH can be explained by the increase in its charge density due to the side chain being positively ionized and adsorbed onto a surface of negative surface charge. This explanation is well suited to the experiments carried out at pH 11, as can be seen in Figure 5D. Arginine is positively charged, and the surfaces are negatively charged. In these systems, it can be thought that electrostatic forces govern the adsorption mechanism. In the case of glycine, as shown in Figure 5A, the amino acid is adsorbed despite having the same surface charge, so the adsorption mechanism must be associated with other processes.

A greatly similar behavior can be observed for lysine and histidine (Figure 5B, Figure 5C, respectively). For aspartic acid, it is observed that when the acidic pH (5.5) is used, the amino acid is negatively charged and the surfaces are positively charged, whereby adsorption could also be due to opposite charge attraction. At the natural pH (Figure 5E), aspartic acid is also in the negative ion form, and it is not adsorbed in any of the surfaces. The differential adsorption of amino acids, even when the charge differences do not exist, may be explain in two different ways. First, it must be considered that the values of the points of zero charge are an average of processes occurring in all the faces of different crystals [67]. Therefore, at the physical scale in which the adsorption process occurs, the surfaces are heterogeneous. On the other hand, at a certain pH value, amino acids become charged (according to their species distribution diagram); however, these changes do not occur simultaneously in all molecules, and there may be some of them, in other ionic forms, that allow interaction with the surface to a lesser extent.

In addition, the constitution of the silicates must be weighed. The building units of silicates are silicon tetrahedrons (SiO_4_), which have a net negative charge. This negative charge must be balanced, and for this silicon, tetrahedrons are associated with positively charged metal ions, such as Fe^2,3+^, Mg^2+^, or Na^+^ [68]. It is with these metal atoms that organic molecules can associate, for example with carboxylic acids through ligands between the oxygen atoms and a metal cation [69]. It has been reported for forsterite (the same variety of olivine that was used in this work) that the Mg^2+^ ions, found on the surface, can interact with water molecules and form H-bonds with the H of the organic molecules and other functional groups thereof [68]. It should be noted that the two proposed scenarios are not mutually exclusive. Due to our results and what has been reviewed in the literature, we suggest that, in addition to electrostatic forces, the adsorption of amino acids may be directed by the formation of organometallic complexes. Specifically for histidine, it has been reported that it can bind to copper (II) metal cations; in these bonds, the metal atoms are able to coordinate up to 3 histidine binding sites, forming complexes of different types. The most determining factor to direct the site and the formation of union between copper and histidine is the pH, which defines the ionic state of the amino acid [70]. The results obtained here are quite interesting, and to corroborate the formation of these complexes it is necessary to carry out more exhaustive analyses. 

In this essay, individual minerals were used to get an idea of the possible contribution of each of them in the sorption of organics, both in the komatiite sample and in the mineral mixture used as a komatiite simulation. However, the results must be treated with care, since the simulated komatiite model represents an approximation of the mineralogical composition of an unaltered komatiite, but not of its structure. Therefore, it does not reproduce important features, such as associations among minerals that compose it, and the interaction between the minerals and the matrix in which they are embedded.

Rocks are in fact heterogeneous adsorbents, real solids that exhibit surface disturbances such as polycrystalline and sometimes amorphous structures, cracks, fissures, dislocation of atoms (due to the presence of impurities), complex porous structure, etc. This surface heterogeneity affects the possible interactions of the solid with the organic molecules, and they vary at different points on the solid [71]. In contrast, all adsorption sites in the surfaces of pure or synthetic solids are considered equivalent, and along the surface they may have a constant and calculable response; in consequence, elementary theories of adsorption can be applied to describe the physical phenomenon occurring on them. However, for a heterogeneous solid, adsorption can be considered as the sum of the independent processes that take place in each phase, as a first approximation. Several attempts have been made to adjust the classical mathematical models of adsorption to describe adsorption in heterogeneous solids, but these solids are very complex systems, and it is also necessary for said mathematical description to have a real physical meaning, which makes it an important challenge [71].

### 4.5. Implications for Prebiotic Chemistry 

On the primitive Earth, as today, the presence of isolated and pure minerals is practically nil. Mineral phases in nature are associated with rock constituents of the continental and oceanic crusts. Considering this, one of the constant criticisms of prebiotic chemistry models is that the simulations performed in the laboratory do not reflect a real scenario. The isolated, pure, or synthetic minerals that are commonly used did not exist on the primitive Earth’s surface. In fact, the mineralogy of a terrestrial planet is considered a highly complex system [72]. Komatiites are the oldest rocks known to date and are believed to have the same composition as the rocks that formed the early oceanic crust. In addition, they can be associated with important primitive environments in prebiotic chemistry such as hydrothermal systems [73].

In these experiments, we wanted to contrast the capacity of adsorption of an altered natural komatiite sample versus a simulated unaltered komatiite. The results showed that the natural komatiite adsorbs more of a diversity of amino acids than the simulation, which is a positive result. The fact that surfaces of natural origin have many peculiarities that allow the adsorption of organic molecules has been much speculated. For example, the topology of the surface, which is a product of the crystallization of the mineral phases and depend on the conditions during the process, is not reproduced. In general, the surfaces of minerals and rocks are irregular or defective; these defects are probable sites where interactions between solids and organics occur [74,75]. In this case, all the characteristics of the komatiite favored the adsorption of organic molecules. The presence of serpentine in the altered komatiite can also be considered as one of the main causes of the differential adsorption between the natural sample and the komatiite-simulated. Said mineral is produced by the alteration of ultramafic rocks, and due to the high hydrothermal activity during the Hadean and Archean eons, serpentines must have been abundant and extremely common [76]. From our point of view, the presence of this alteration emulates more real conditions of the complex concentrator surfaces present in possible primitive scenarios. These results highlight the importance of further studies to analyze the concentrating role of serpentines. These complex surfaces are very difficult to study; despite this, it is important to understand their possible contribution to prebiotic processes. In addition to this, there is a particular interest in studying olivine since it is found not only on the surface of the Earth, but is also a mineral found in interstellar and interplanetary dust particles, as well as being present in meteorites and comets [68]. It can also be mentioned that forsterite is a relevant material for many environments, including Martian-type samples [77].

## 5. Conclusions

In this work, we studied the adsorption of five different amino acids (glycine, lysine, histidine, arginine and aspartic acid) on (1) a natural komatiite, (2) a simulated komatiite, which is a solid mixture of the three main constitutive minerals (olivine, pyroxene, and plagioclase), and (3) pure separates of each mineral. To do so, we explored the relevance of pH changes in the adsorption process.

There is a differential adsorption of the amino acids onto the solids. In general, amino acids are better adsorbed at alkaline conditions, regardless of their nature (either hydrophobic or hydrophilic) or the surface employed. This could be relevant in a prebiotic context, even when there is no consensus on the pH of primitive oceans, because a high pH would prevail in white smoker-like hydrothermal systems.

The amino acids histidine and arginine were adsorbed on most surfaces and under different pH conditions. The relevance of this finding relies on the fundamental role that these amino acids play in the stabilization of oligomers or as constituents of catalytic oligomers.

The adsorption mechanism of the organics is complex. The charge of the molecule (in the function of pH) turned out to be an important factor in the adsorption of amino acids since they could be adsorbed on surfaces by opposite charge attraction. However, electrostatic forces do not fully explain the observed adsorption in all systems. Other possible key factors in governing adsorption, associated with differences in adsorption, are the length and the functional groups of the side chain of the amino acids. Another relevant phenomenon for the matter is the formation of the sphere of solvation, which surrounds the amino acids in aqueous solution and contributes to the stabilization of the molecule. Further, the heterogeneity of surfaces and their chemical composition, in particular the number of cations on the surfaces, have a very important effect in sorption, as the occurrence cations is key in the formation of organometallic compounds.

Natural komatiite is the surface where the adsorption of most amino acids occurred, at different pH values. This is relevant since natural adsorption would have occurred on rocks, which are constituted by complex mineral interrelations. Individual minerals also adsorb amino acids, and the analyzed plagioclase is the mineral onto which more amino acids (at different pH) were adsorbed.

This work represents an effort to use komatiites as complex surfaces to study sorption processes. The results shown constitute a first approximation of further studies by using materials that recreate the likeliest substrates for prebiotic reactions. Our future experiments will include aqueous solutions with different concentrations of salts (simulating the composition of primitive oceans), and the use of mixtures of organics, in order to construct models that are closely related to prebiotic environments. 

## Figures and Tables

**Table 1:** The main chemical properties of the five amino acids used.

Amino Acid	Classification	Formula	p*K*a			pI
			p*K*_1_	p*K*_2_	p*K*_3_	
Gly	Neutral	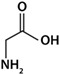	2.34	9.60	-	5.98
Lys	Basic	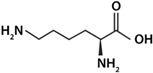	2.18	8.95	10.53	9.74
His	Basic	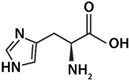	1.82	9.17	6.00	7.59
Arg	Basic	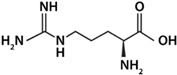	2.17	9.04	12.48	10.76
Asp	Acid	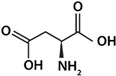	1.88	9.60	3.65	2.77

**Table 2:** Experimental pH values, determined for each amino acid in a solution without further modification.

Amino Acid	pH Value
Gly	5.8 ± 0.51
Lys	7.3 ± 0.26
His	6.7 ± 0.23
Arg	8.23 ± 0.6
Asp	4.3 ± 0.28

Note: The values presented here are the average of five independent measurements.

**Table 3:** Mineral phases and the semiquantitative percentage of each one, found in the komatiite sample, all determined by X-ray diffraction analysis by the Reference Intensity Radio (RIR) method.

Mineral Phases	SemiQuant RIR (%)
Olivine (forsterite: Mg_2_SiO_4_)	10
Pyroxene (diopside: CaMgSi_2_O_8_)	14
Plagioclase (bytownite: (NaCa)AlSi_3_O_8_)	26
Serpentine (lizardite:Mg_3_Si_2_O_5_(OH)_4_)	29
Other phyllosilicates ~14 Å ppb type of chlorite	21
Total	100

**Table 4:** Estimated *pzc* values for geological materials used.

Geological Materials	*pzc*
Olivine	9.9
Pyroxene	7.5
Plagioclase	8.9
Komatiite	8.2
Komatiite-simulated *Olivine 49.29%* *Plagioclase 29.57%* *Pyroxene 21.12%*	10.05

Note: The komatiite-simulated sample is a solid mixture of the minerals named.

**Table 5:** Number of amino acids adsorbed in each geological material; the pH of the solutions was not modified; see Table 2 for reference.

Geological Materials	Adsorption (mmol/mg)				
	Gly ± SD	Lys ± SD	His ± SD	Arg ± SD	Asp
Olivine	3.18 × 10^−5^ ± 2.2 × 10^−6^	1.27 × 10^−4^ ± 4.9 × 10^−6^	7.17 × 10^−5^ ± 1.9 × 10^−6^	2.11 × 10^−4^ ± 5.1 × 10^−6^	-
Pyroxene	2.31 × 10^−4^ ± 7 × 10^−6^	1.12 × 10^−4^ ± 2 × 10^−6^	-	-	-
Plagioclase	1.55 × 10^−5^ ± 1.4 × 10^−7^	1.41 × 10^−4^ ± 9.8 × 10^−6^	2.15 × 10^−5^ ± 2.1 × 10^−8^	1.22 × 10^−4^ ± 1.8 × 10^−7^	-
Komatiite	6.14 × 10^−4^ ± 3.3 × 10^−6^	6.99 × 10^−4^ ± 4.3 × 10^−5^	2.64 × 10^−4^ ± 1.0 × 10^−6^	8.76 × 10^−4^ ± 1.4 × 10^−4^	-
Komatiite-simulated	-	-	-	5.14 × 10^−5^ ± 4.1 × 10^−7^	-

Note: Amounts are presented as millimoles of amino acid adsorbed per milligram of solid used. The calculated standard deviation of the measurements is shown after the sorption values (± SD). For experiments in which adsorption was not observed, a dash (-) symbol is used.

**Table 6:** Adsorbed quantities of amino acids in each geological material, at acidic pH (5.5).

Geological Materials	Adsorption (mmol/mg)				
	Gly ± SD	Lys ± SD	His ± SD	Arg ± SD	Asp ± SD
Olivine	-	-	4.52 × 10^−5^ ± 3.8 × 10^−6^	9.55 × 10^−5^ ± 1 × 10^−6^	1.52 × 10^−4^ ± 1.4 × 10^−6^
Pyroxene	4.74 × 10^−6^ ± 1.9 × 10^−8^	-	1.75 × 10^−4^ ± 4.9 × 10^−6^	8.94 × 10^−5^ ± 1.8 × 10^−7^	1.27 × 10^−4^ ± 2.1 × 10^−6^
Plagioclase	-	-	9.40 × 10^−4^ ± 1.2 × 10^−5^	8.93 × 10^−5^ ± 2.7 × 10^−7^	9.00 × 10^−5^ ± 5.4 × 10^−7^
Komatiite	-	1.69 × 10^−4^ ± 4.6 × 10^−6^	2.85 × 10^−4^ ± 4.2 × 10^−5^	3.96 × 10^−4^ ± 2.4 × 10^−6^	2.57 × 10^−4^ ± 1 × 10^−5^
Komatiite-simulated	1.65 × 10^−4^ ± 3.3 × 10^−6^	-	2.44 × 10^−4^ ± 2.5 × 10^−6^	1.72 × 10^−4^ ± 4.1 × 10^−6^	1.21 × 10^−4^ ± 4.5 × 10^−6^

Note: Amounts are presented as millimoles of amino acid adsorbed per milligram of solid used. The calculated standard deviation of the measurements is shown after the sorption values (± SD). For experiments in which adsorption was not observed, a dash (-) is used.

**Table 7:** Adsorbed quantities of amino acids in each geological material, at alkaline pH (11).

Geological Materials	Adsorption (mmol/mg)				
	Gly ± SD	Lys ± SD	His ± SD	Arg ± SD	Asp ± SD
Olivine	6.90 × 10^−5^ ± 2.6 × 10^−6^	1.08 × 10^−4^ ± 2.1 × 10^−6^	6.52 × 10^−5^ ± 2.7 × 10^−6^	3.40 × 10^−4^ ± 1.3 × 10^−5^	1.04 × 10^−5^ ± 3.5 × 10^−7^
Pyroxene	3.18 × 10^−5^ ± 3.4 × 10^−7^	1.35 × 10^−4^ ± 1.5 × 10^−6^	-	2.15 × 10^−4^ ± 1.6 × 10^−6^	1.34 × 10^−4^ ± 2.7 × 10^−6^
Plagioclase	4.68 × 10^−5^ ± 1.4 × 10^−6^	1.90 × 10^−4^ ± 1.2 × 10^−5^	1.72 × 10^−4^ ± 1.1 × 10^−5^	2.19 × 10^−4^ ± 9.3 × 10^−6^	5.29 × 10^−5^ ± 1.7 × 10^−6^
Komatiite	2.39 × 10^−5^ ± 2.9 × 10^−7^	4.76 × 10^−4^ ± 1.5 × 10^−5^	2.91 × 10^−5^ ± 3 × 10^−7^	3.82 × 10^−4^ ± 8.8 × 10^−5^	5.70 × 10^−7^ ± 2.7 × 10^−8^
Komatiite-simulated	2.27 × 10^−4^ ± 4.5 × 10^−6^	2.12 × 10^−4^ ± 3.6 × 10^−6^	6.45 × 10^−5^ ± 2.3 × 10^−6^	1.50 × 10^−4^ ± 3.3 × 10^−5^	5.57 × 10^−5^ ± 6.8 × 10^−7^

Note: Amounts are presented as millimoles of amino acid adsorbed per milligram of solid used. The calculated standard deviation of the measurements is shown after the sorption values (± SD). For experiments in which adsorption was not observed, a dash (-) is used.

## Data Availability

Not applicable.
